# Longitudinal and Comparative Analysis of Gut Microbiota of Tunisian Newborns According to Delivery Mode

**DOI:** 10.3389/fmicb.2022.780568

**Published:** 2022-04-25

**Authors:** Mariem Hanachi, Olfa Maghrebi, Haifa Bichiou, Ferdaous Trabelsi, Najla Maha Bouyahia, Fethi Zhioua, Meriam Belghith, Emna Harigua-Souiai, Meriem Baouendi, Lamia Guizani-Tabbane, Alia Benkahla, Oussema Souiai

**Affiliations:** ^1^Laboratory of Bioinformatics, bioMathematics and Biostatistics—LR16IPT09, Institut Pasteur de Tunis, Université de Tunis El Manar, Tunis, Tunisia; ^2^Faculty of Science of Bizerte, University of Carthage, Tunis, Tunisia; ^3^Laboratory of Transmission, Control, and Immunobiology of Infections—LR16 IPT02, Institut Pasteur de Tunis, Université de Tunis El Manar, Tunis, Tunisia; ^4^Laboratory of Medical Parasitology, Biotechnology, and Biomolecules—LR16 IPT06, Institut Pasteur de Tunis, Université de Tunis El Manar, Tunis, Tunisia; ^5^Service de Gynécologie et Obstétrique, Hôpital Régional de Zaghouan, Zaghouan, Tunisia; ^6^Service de Gynécologie Obstétrique et Médecine de la Reproduction, Hôpital Aziza Othmana, Tunis, Tunisia; ^7^Laboratory of Molecular Epidemiology and Experimental Pathology—LR16IPT04, Institut Pasteur de Tunis, Université de Tunis El Manar, Tunis, Tunisia

**Keywords:** shotgun metagenome sequencing, newborns, elective cesarean deliveries, ESKAPE bacteria, Tunisia, microbiome

## Abstract

Microbiota colonization is a dynamic process that impacts the health status during an individual's lifetime. The composition of the gut microbiota of newborns is conditioned by multiple factors, including the delivery mode (DM). Nonetheless, the DM's influence remains uncertain and is still the subject of debate. In this context, the medical indication and the emergency of a cesarean delivery might have led to confounding conclusions regarding the composition and diversity of the neonatal microbiome. Herein, we used high-resolution shotgun sequencing to decipher the composition and dynamics of the gut microbiota composition of Tunisian newborns. Stool samples were collected from 5 elective cesarean section (ECS) and 5 vaginally delivered (VD) newborns at the following time points: Day 0, Day 15, and Day 30. The ECS and VD newborns showed the same level of bacterial richness and diversity. In addition, our data pointed to a shift in microbiota community composition during the first 2 weeks, regardless of the DM. Both ECS and VD showed a profile dominated by Proteobacteria, Actinobacteria, and Firmicutes. However, ECS showed an underrepresentation of *Bacteroides* and an enrichment of opportunistic pathogenic species of the ESKAPE group, starting from the second week. Besides revealing the intestinal microbiota of Tunisian newborns, this study provides novel insights into the microbiota perturbations caused by ECS.

## Introduction

The bacterial colonization of the gut microbiota has a tremendous impact on health across the lifespan (Houghteling and Walker, 2015; Walker, 2017). Although suspected to be initiated *in utero*, microbiota colonization is a highly fluctuant and dynamic process during the first days of life (Perez-Muñoz et al., 2017; Walker, 2017; Sanidad and Zeng, 2020). This process occurs in successive stages, leading to a stable adult-like profile by the age of 3 years (Yatsunenko et al., 2012; Walker, 2017; Sanidad and Zeng, 2020). Pioneer bacteria that colonize the newborn's intestinal tract are essential for immune system maturation, gastrointestinal metabolism, and neurological development (Borre et al., 2014; Milani et al., 2017; Stiemsma and Michels, 2018; Sanidad and Zeng, 2020).

Most microbiome research has focused on Western countries, and only marginal analyses were interested in low-income African countries (Hamdi et al., 2021). To our knowledge, none concerned North Africa, despite the well-established effect of geographical localization and population habits on microbiome composition and diversity (Fallani et al., 2010; Yatsunenko et al., 2012). More specifically, Tunisia constitutes a particular case study, considering the dramatically high rate of (43%) cesarean section (CS) compared with thresholds established by the World Health Organization (WHO) (Unicef report, 2018).

Among all influencing factors, the delivery mode (DM) was extensively investigated and is still subject to various controversies and debates (Aagaard et al., 2016; Stinson et al., 2018). The first high throughput microbiome analyses focusing on this topic conducted by Dominguez-Bello et al. reported that the gut microbiota of the vaginally delivered (VD) newborns is similar to mother vaginal microbiota, while the microbiota of CS newborns resembles the maternal skin microbiota (Dominguez-Bello et al., 2010). Limited and altered diversity was subsequently described for CS delivered newborns (Rutayisire et al., 2016; Shi et al., 2018). These observations have been linked to a higher susceptibility to develop immune and metabolic disorders observed in CS infants (Darmasseelane et al., 2014; Sevelsted et al., 2015; Wampach et al., 2018; Busi et al., 2021). In spite of this established effect of DM on infant health, the consensus on the immature status of the CS delivery microbiome has been contradicted by some studies reporting no effects on newborn's microbiota neither in terms of composition nor in terms of functionality (Hu et al., 2013; Chu et al., 2017; Liu et al., 2019). It was suggested that the reported differences might be biased by confounding factors such as intrapartum antibiotic administration, differences in breastfeeding behaviors, gestational age, and like in our case of interest, the indication for CS (Aagaard et al., 2016; Chu et al., 2017; Stinson et al., 2018).

In fact, emergent and elective CS (ECS) differs in several aspects that may affect the maternal and newborn microbiota. First, ECS is undertaken without rupture of the fetal membranes, and newborns are delivered without passing through the birth canal. They are thereby not exposed to the maternal vaginal flora (Stinson et al., 2018). Second, emergent CS occurs after the start of labor and is characterized by a fluctuation in levels of endocrine, pro-inflammatory, and immune-mediating cytokines, in opposition to ECS (Malamitsi-Puchner et al., 2005; Francino, 2018; Stinson et al., 2018). Thus, ECS constitutes a unique opportunity to further assess the real impact of DM on the gut microbiota of newborns, as it prevents the contact of the newborn with the maternal vaginal flora during birth and the potential biases due to the medical indication of emergent CS.

Altogether, the arguments listed above raised our interest to first uncover the microbiota of Tunisian newborns and then to assess the impact of DM on the composition of early gut bacteria by minimizing confounding factors. A shotgun metagenomic approach was applied to better capture the microbial resolution in the ECS and VD newborns.

## Materials and Methods

### Ethics Statements

This study was approved by the Biomedical Ethics Committee of the Institute Pasteur of Tunis, under reference number 2018/10/I/LR161IPT09. Written informed consents were obtained from both parents.

### Participants' Recruitment and Sample Collection

All participants originated from Zaghouan, a semirural region in Northwest Tunisia. The mothers had a non-declared pathology and did not receive antibiotics, anti-inflammatory drugs, or immunosuppressive therapy during the last trimester of pregnancy. Neonates were healthy, full-term born (>37 weeks), and did not receive any antibiotic treatment. Only newborns delivered by elective and unlabored cesarean were included in this study. No complications during the delivery were reported. Anonymized mother's health records with their corresponding metadata were compiled. Clinical metadata concerning the newborns were also collected including birth weight, gestational age, gender, weight, and feeding mode (Table 1).

**Table 1:** Clinical characteristics of the newborns and mothers enrolled in this study.

	**ECS (*N* = 5)**	**VD (*N* = 5)**	**Total (*N* = 10)**	***p*-value**
**Gender**				0.197^a^
Female	3 (60.0%)	1 (20.0%)	4 (40.0%)	
Male	2 (40.0%)	4 (80.0%)	6 (60.0%)	
**Gestational age (years)**				0.059^b^
Mean (SD)	39.166 (0.894)	37.740 (1.141)	38.453 (1.224)	
Range	37.710–40.140	36.280–39	36.280–40.140	
**Newborns weight (g)**				0.092^a^
Mean (SD)	3,600 (367.423)	3,246 (191.259)	3,423 (333.268)	
Range	3,000–4,000	3,080–3,500	3,000–4,000	
**Feeding mode [Day 0–Day 15]**				0.368^a^
N-miss	1 (20.0%)	0 (0.0%)	1 (10.0%)	
Breastfed	4 (80.0%)	4 (80.0%)	8 (80.0%)	
Mixed feeding	0 (0.0%)	1 (20.0%)	1 (10.0%	
**Feeding mode [Day 15–Day 30]**				0.368^a^
(Missing)	1 (20.0%)	1 (20.0%)	2 (20.0%)	
Breastfed	3 (60.0%)	1 (20.0%)	4 (40.0%)	
Mixed feeding	1 (20.0%)	3 (60.0%)	4 (40.0%)	
**Feeding mode [Day 0–Day 30]**				0.506^a^
N-Miss	1 (20.0%)	1 (20.0%)	2 (20.0%)	
Exclusively breastfed	3 (60.0%)	1 (20.0%)	4 (40.0%)	
Not breastfed	0 (0.0%)	1 (20.0%)	1 (10.0%)	
Mixed breastfed	1 (20.0%)	2 (40.0%)	3 (30.0%)	
**Mothers weight after.pregnancy (g)**				0.892^b^
N-Miss	1	0	1	
Mean (SD)	71.250 (7.500)	70.200 (13.236)	70.667 (10.440)	
Range	64–81	58–85	58–85	
**Mothers age (years)**				0.854^b^
Mean (SD)	31.200 (5.848)	30.600 (3.912)	30.900 (4.701)	
Range	23–39	24–34	23–39	
**Primiparity**				0.490^b^
No	3 (60.0%)	4 (80.0%)	7 (70.0%)	
Yes	2 (40.0%)	1 (20.0%)	3 (30.0%)	

*The tests used to calculate p-values differ by the variable type*:

a *Pearson's chi-squared test*;

b *linear model ANOVA*.

A total of 10 participants were retained, and newborns' stool samples were collected at three time points. The neonatal meconium from VD and ECS newborns was collected during the first 24 h (Day 0) at the regional hospital of Zaghouan and at the Aziza Othmana hospital, located in Zaghouan and Tunis, respectively. The collection was renewed for 2 weeks (Day 15) and 1 month (Day 30) after birth. The samples were transported in ice bags to the laboratory and then stored at −80°C until DNA extraction.

### DNA Extraction and Sequencing

Subsamples from each newborn stool (~200 mg) were used for DNA extraction. The solubilization and liquefaction of the samples were carried out using 1 ml of Tween 20 (10%). The solution was then vortexed horizontally for 20 min and centrifuged at 14,000 rpm for 10 min at 4°C. The obtained pellets were resuspended in a lysis buffer (100 mM NaCl, 25 mM EDTA, 0.5% SDS, 10 mM HEPES, and 1 mg/ml lysozyme) for chemical and enzymatic lysis. Then, bead-beating steps were proceeded using BeadBug and PowerBead Tubes, Garnet 0.70 mm (QIAGEN), with a setting of 3 × 20 s pulses, followed by incubation for 7 min at 95°C. After a centrifugation step, the supernatant was transferred into 2 ml tubes containing 30 μl proteinase K and 400 μl AL buffer (QIAamp Fast DNA Stool Mini Kit) and incubated at 70°C for 10 min. DNA was recovered using phenol-chloroform extraction and purified using the QIAamp DNA mini kit (QIAGEN). In brief, protein precipitation was performed using the phenol/chloroform method, followed by DNA precipitation using absolute ethanol. The supernatant was transferred to the QIAamp spin column, and DNA purification was performed according to the QIAamp Fast DNA Stool Mini Kit manufacturer's instructions. DNA samples were quantified using the Multiskan™ GO Microplate Spectrophotometer (Thermo Fisher™), and samples with >100 ng/μl DNA material proceeded to the paired-end (2 × 150 bp) metagenomic sequencing. Overall, we sequenced 33 samples: 9 participants at 3 time points, 1 participant at 2 time points (Day 0 and Day 30), and 4 technical replicates (2 at Day 0, 1 at Day 15, and 1 at Day 30).

### Bioinformatics and Biostatistics Analyses

Quality control and preprocessing were undertaken on the raw data as follows: Trimmomatic was run to trim low-quality bases and short reads (SLIDINGWINDOW: 4:20, min length: 70) (Bolger et al., 2014). Following this step, we obtained on average 11 and 14 M reads for Day 15 and Day 30 samples. As expected, Day 0 samples harbored fewer sequences, 28.6 K reads on average (Supplementary Table 1). Then, human DNA and vector contaminants were screened and removed using FastQ Screen software (Wingett and Andrews, 2018). The taxonomic classification of cleaned metagenomic reads was performed using Kraken version 2.0.8 against the Minikraken Database (Wood et al., 2019). Then, Bracken version 2.5 was run on the Kraken taxonomic assignment outputs to increase the accuracy of the abundance estimates at species and genus levels (Lu et al., 2017).

The classification output consisted of a feature table containing 3,805 taxa composed of bacteria 3,489 operational taxonomic units (OTUs) as well as viruses (212 OTUs) and Archaea (103 OTUs). The statistical analysis started with filtering ambiguous and rare bacterial taxa (Supplementary Table 1). First, low-prevalent (<15% samples) and low abundant (<100 reads) phyla were removed. OTUs present in <15% of samples were also excluded. None of the filtered OTUs were specific to a mode of delivery or unique to collection day, thus not impacting the overall rates of abundance (data not shown). Ultimately, 2,609 OTUs belonging to bacteria remained in the taxonomy table.

Alpha diversity metrics (Shannon and Simpson indices and OTU richness) were analyzed using the microbiome R package (McMurdie and Holmes 2013).

A phylogenetic tree was generated and used to estimate Faith phylogenetic diversity (standardized effect sizes = TRUE) using the function “phyloseq_phylo_div” from the R package PhyloMeasures (Tsirogiannis and Sandel, 2016) and metagMisc R package (available at: https://github.com/vmikk/metagMisc). The Friedman test was used to compare differences in alpha diversity between collection days (paired samples), followed by the Nemenyi *post-hoc* test using the PMCMRplus R package (available at https://cran.r-project.org/web/packages/PMCMRplus/index.html). When comparing alpha diversity between two unpaired groups, the non-parametric Wilcoxon was employed. Kruskal–Wallis test was used to assess statistical differences between more than two groups, followed by *post-hoc* Dunn's test (with Bonferroni correction). The clustering and heatmap were performed using the microbiomeutilities R package (Shetty and Lahti, 2020). The clustering based on taxa and samples was performed using Bray–Curtis dissimilarity distances.

Beta diversity was evaluated with the Bray–Curtis distance after data normalization using the Cumulative Sum Scaling (CSS) (Paulson et al., 2013). Visualization was performed using the Non-metric Multidimensional Scaling (NMDS) ordination method. Differences between the groups were tested using the permutational multivariate analysis of variance (PERMANOVA) test (number of the permutation set to 999). The linear discriminant analysis effect size (LEfSe) R package was used to identify the differentially abundant taxa between study groups on CSS normalized data (Segata et al., 2011) (parameters: kw_cutoff = 0.01, wilcoxon_cutoff = 0.01, and lda_cutoff = 3). PERMANOVA test was also run to determine covariate impact on newborns' gut microbial community based on Bray–Curtis distance (permutation set to 999).

Functional analysis was processed with SqueezeMeta Pipeline using the merged mode (Tamames and Puente-Sánchez, 2019). First, Megahit was used for the assembly (—min-count 1—k-list 27, 37, 47, 57, 67, 77, 87) (Li et al., 2015). We obtained 139,759 contigs ranging from 200 to 761,139 bp (Supplementary Table 1). Then, open reading frame (ORF)'s prediction and Kyoto Encyclopedia of Genes and Genomes (KEGG) pathway (KEGG database, release 95, July 2020) prediction were performed by Prodigal and Diamond, respectively (Hyatt et al., 2010; Buchfink et al., 2015). Coverage and abundance estimation for genes and contigs were performed using Bedtools and normalized using the reads per kilobase per million mapped reads (RPKM) method (Quinlan and Hall, 2010). The statistical downstream analyses were performed using the SQMtools and phyloseq R packages (McMurdie and Holmes, 2013; Puente-Sánchez et al., 2020).

## Results

### Characteristics of the Participants

Full-term newborns (5 from vaginal delivery and 5 from ECS delivery) were delivered at a mean gestational age of 38.5 weeks (SD ± 1.22). Samples were collected on the birthday (Day 0), Day 15, and Day 30 after birth. The characteristics of the mothers and newborns are listed in Table 1. Overall, 40% of newborns were exclusively breastfed, 10% exclusively received formula milk, and 30% received mixed feeding. Notably, 80% of neonates were breastfed until the second time point (Day 15). By Day 30, this percentage decreased to 40%. There were no significant differences in maternal age [31 years (SD ± 4.70)], mother's weight [70.76 kg (SD ± 10.44)], and birth weight [3.43 kg (SD ± 0.34)] between ECS and VD (Table 1).

### Composition of Gut Microbiota of the Tunisian Newborns in the Early Stage of Life

We first aimed to determine the significant factors associated with microbiota structure. Therefore, we performed the PERMANOVA test individually for each covariate. The results showed that collection days explained most of the variance in population structure (Supplementary Table 2; *p* = 0.001, *R*^2^ = 0.210). Although not impacting the diversity, feeding mode was the second most significant factor explaining variation in neonatal gut microbiota (*p* = 0.033, *R*^2^ = 0.115; Supplementary Figure 1, Supplementary Table 2). The other clinical covariates (newborns' weight and gestational age) did not influence the community structure of the early microbiome (Supplementary Table 2).

**Figure 1:**
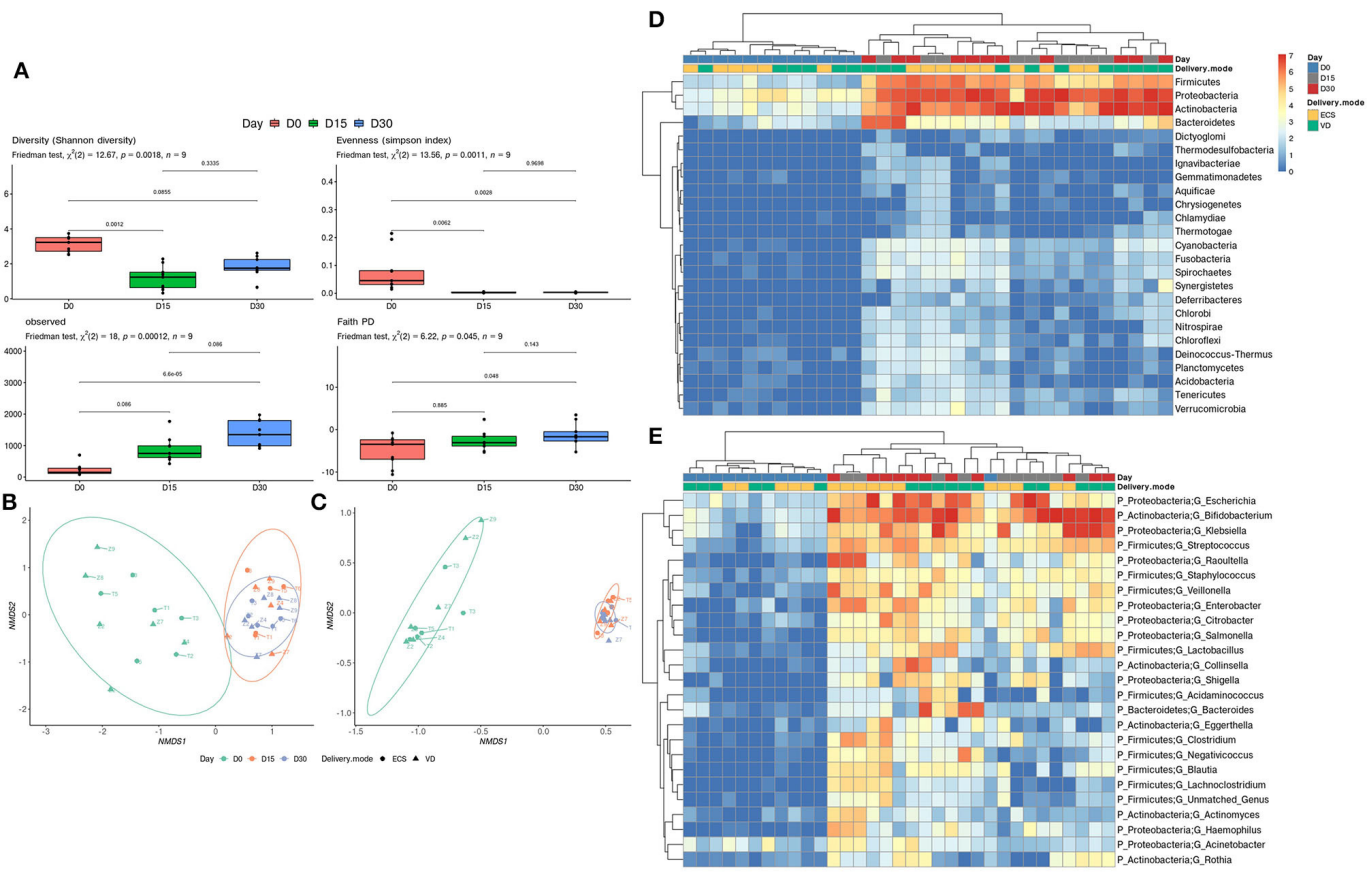
Development of early gut microbiota of Tunisian newborns. **(A)** Alpha diversity was measured by estimating the Shannon index (top right panel), Simpson index (top left panel), observed OTU richness (bottom left panel), and Faith phylogenetic diversity (bottom right panel). Significant differences between groups were determined using the Friedman test followed by a Nemenyi *post-hoc* test. Ordination plot with nonmetric multidimensional scaling (NMDS) based on the relative abundance of taxa **(B)** and pathways **(C)** in newborns' gut microbiota. Heatmap of relative abundance (log_10_ transformed) of the 25 most abundant taxa at the phylum **(D)** and genus level **(E)**. The clustering was performed based on Bray–Curtis distance measure.

We then scrutinized the early establishment of Tunisian newborns' gut microbiota by comparing the three sampling time points. This allowed us to identify the temporal patterns in terms of taxonomic diversity. A significant decrease in terms of Shannon evenness and Simpson diversity indices was observed when comparing Day 0 with Day 15 and Day 30 (Figure 1A, top left and top right panel). In addition, the number of observed OTUs and phylogenetic diversity significantly increases over time (Figure 1A, bottom left and bottom right panel). The beta-diversity analysis clearly and significantly distinguished the Day 0 samples from the other time points (Day 15 and Day 30) (*p* = 0.001, *R*^2^ = 0.210; Figure 1B). The functional annotation analysis corroborated this ascertainment (Figure 1C; *p* = 0.001, *R*^2^ = 0.688). This suggests that the functions potentially brought by bacteria at Day 0 are distinct from those ensured at Day 15 and Day 30. At the phylum level, a pattern of increasing abundance composed of Firmicutes, Proteobacteria, and Actinobacteria is observed (Figure 1D). Clustering at the genus level revealed that the abundance of *Escherichia, Klebsiella* from the Proteobacteria, *Bifidobacterium* from the Actinobacteria, and *Streptococcus* from the Firmicutes increases from Day 15 post-delivery (Figure 1E). The LEfSe analysis highlighted a significant increase for the above-mentioned genera and phyla (Supplementary Table 3).

### DM Impacts the Distribution of Key Taxa in Newborns' Gut Microbiota

**Figure 2:**
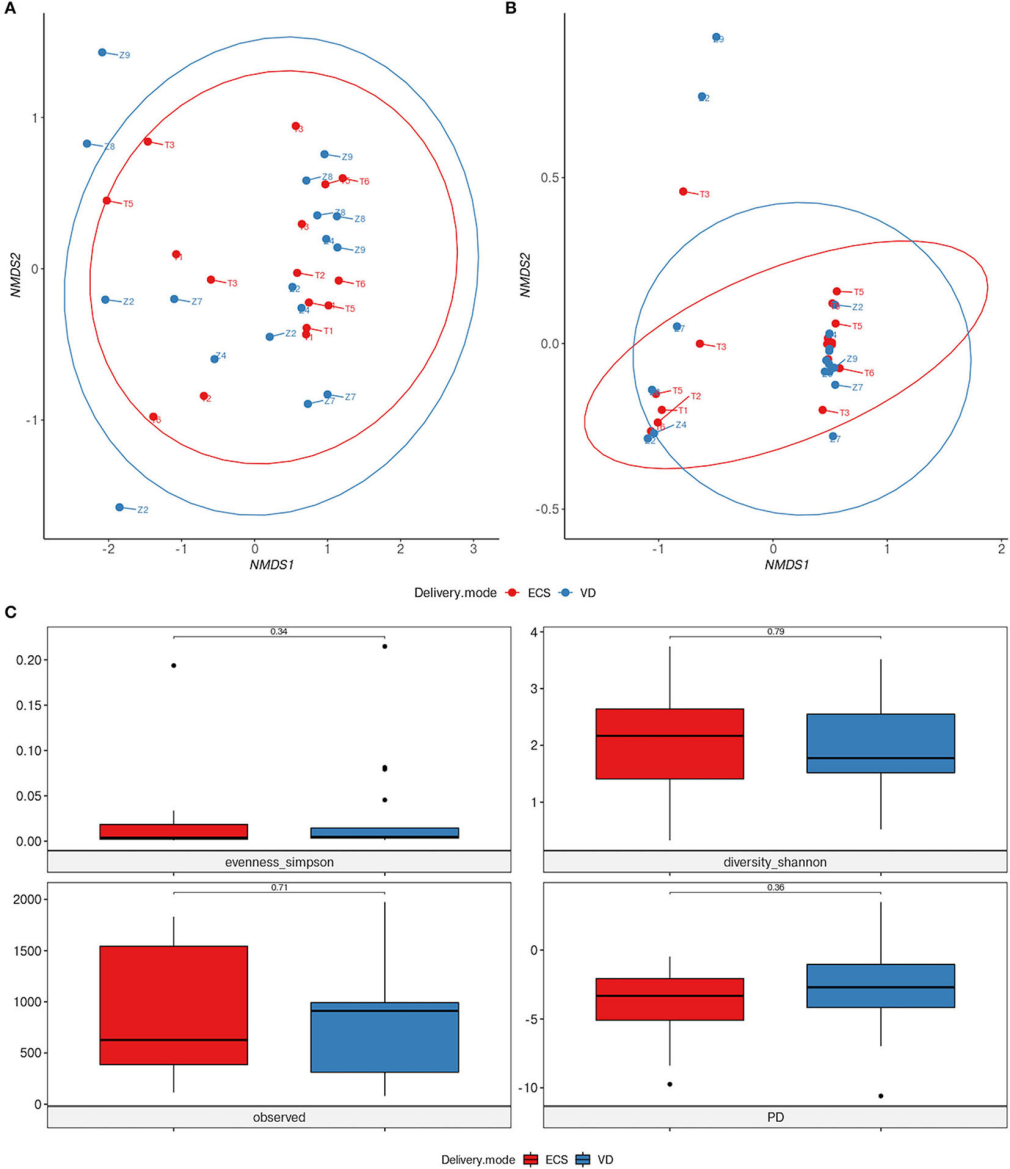
Impact of vaginal and elective cesarean on the diversity of the early life gut microbiome. NMDS ordination plot based on the relative abundance of taxa **(A)** and pathways **(B)** in newborns' gut microbiota. Samples are marked according to delivery mode (DM). **(C)** Alpha diversity was measured by Shannon and Simpson indices (panel top), observed OTU richness (panel bottom left), and Faith phylogenetic diversity (panel bottom right). Significant differences between groups were determined using a Mann–Whitney *U*-test.

To address the impact of DM on newborns' gut microbiota, we carried out various taxonomic and functional diversity analyzes. Alpha and beta-diversity showed no significant difference neither in terms of diversity nor in bacterial community structure between ECS and VD groups (*p* = 0.331, *R*^2^ = 0.034) (Figures 2A,C). Similarly, we did not observe differences in community function between ECS and VD (Figure 2B; *p*-value = 0.75, *R*^2^ = 0.011).

Although the ECS and VD microbiota shares a similar community structure, marginal taxonomic differences do exist. To further characterize these differences, LEfSe analysis was performed to detect specific differentially abundant features at various taxonomic ranks in ECS and VD (Supplementary Figure 2). In the ECS group, species belonging to *Actinomyces, Pseudopropionibacterium*, and *Citrobacter* genera were overrepresented compared with the VD group. Interestingly, the microbiota of ECS newborns was enriched in the genus *Clostridium*. Among these genera, the *Clostridium perfringens*, an opportunistic pathogenic species, was detected as overrepresented. In addition, the ECS showed an overrepresentation of *Enterobacter* species, a highly virulent and antibiotic-resistant member of the ESKAPE bacterial group.

The Bacteroidetes phylum, including species belonging to the *Bacteroides* genus (*Bacteroides helcogenes*; *Bacteroides heparinolyticus*), and *Parabacteroides* were identified as overrepresented in the VD group (Supplementary Figure 2). The ECS group also showed an enrichment in the *Bifidobacterium dentium*, while the *Bifidobacterium pseudocatenulatum* was overrepresented in the VD group.

Taken together, these results indicate that although the mode of delivery does not apparently impact the overall diversity, there is nevertheless a significant difference at phylum and genus level between the two groups. These differences are mirrored by the enrichment of opportunistic pathogenic species in ECS and *Bacteroidetes* in VD.

### The Establishment of Gut Microbiota in ECS and VD Newborns Exhibits Different Dynamics

**Figure 3:**
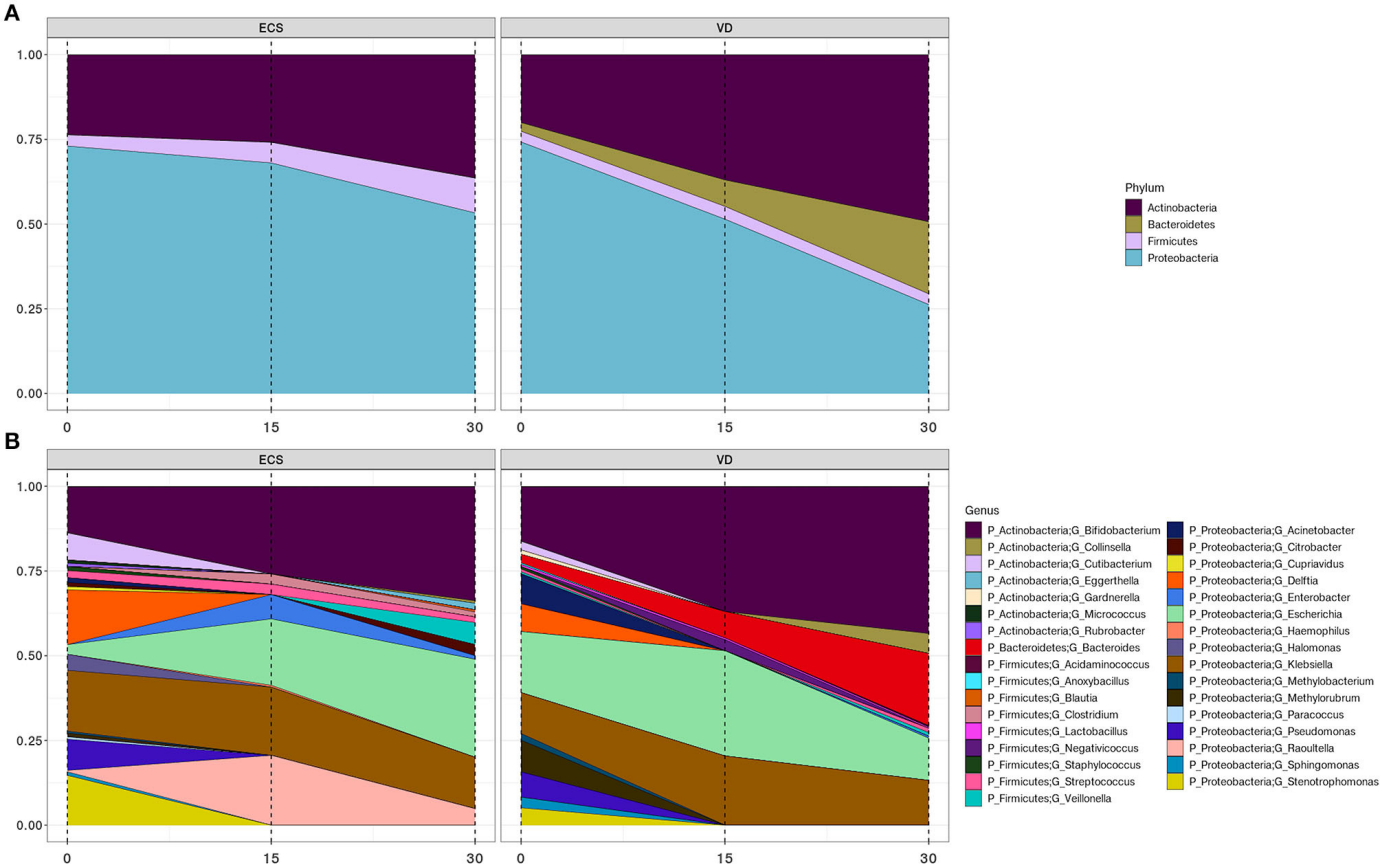
Development trajectory of the newborn's gut microbiota according to DM. Area plots showing the longitudinal changes of >2% relative abundance across all samples of bacterial phyla **(A)** and their corresponding genera **(B)** in elective cesarean section (ECS) and vaginally delivered (VD).

We then sought to understand how the composition and developmental trajectory of the early gut microbiota occurs in ECS and VD. A transition that occurs within the first 2 weeks of life was observed in both ECS (*p* = 0.002, *R*^2^ = 0.246) and VD (*p* = 0.002, *R*^2^ = 0.269; Figure 3).

While the gut microbiota of ECS neonates was colonized by Actinobacteria, Proteobacteria, and Firmicutes, the microbiota of the VD group also included the Bacteroidetes phylum. The Bacteroidetes were represented exclusively by the genus *Bacteroides*, while the genus *Raoultella* was strictly observed in ECS neonates (Figures 3A,B, Supplementary Table 4). We also observed a gradual increase of Actinobacteria at the expense of Proteobacteria (Figure 3A). This trend appears to be a consequence of the progressive growth of the genus *Bifidobacterium*, at a lower rate in ECS, and the decline of the genera *Delftia, Enterobacter, Sphingomonas*, and *Stenotrophomonas* (Figure 3B, Supplementary Table 3). With a lower relative abundance, the Firmicutes remain constant in the ECS while increasing in the VD, possibly due to expansion of the *Veillonella* genus (Figures 3A,B, Supplementary Table 4). At the genus level, the most striking difference between the two groups is the presence of *Lactobacillus* and *Collinsella* in VD.

Some genera present at birth do not persist in the remaining time points. This was observed for *Cutibacterium, Acinetobacter, Delftia, Pseudomonas, Sphingomonas, Stenotrophomonas*, and *Methylobacterium*, regardless of DM. While focusing on ECS newborns' meconium, we noticed that genera *Citrobacter, Cupriavidus, Halomonas*, and *Paracoccus* are undetected from Day 15. Similarly, *Gardnerella* and *Methylorubrum* are solely present in VD newborns' meconium during the first 2 weeks.

**Figure 4:**
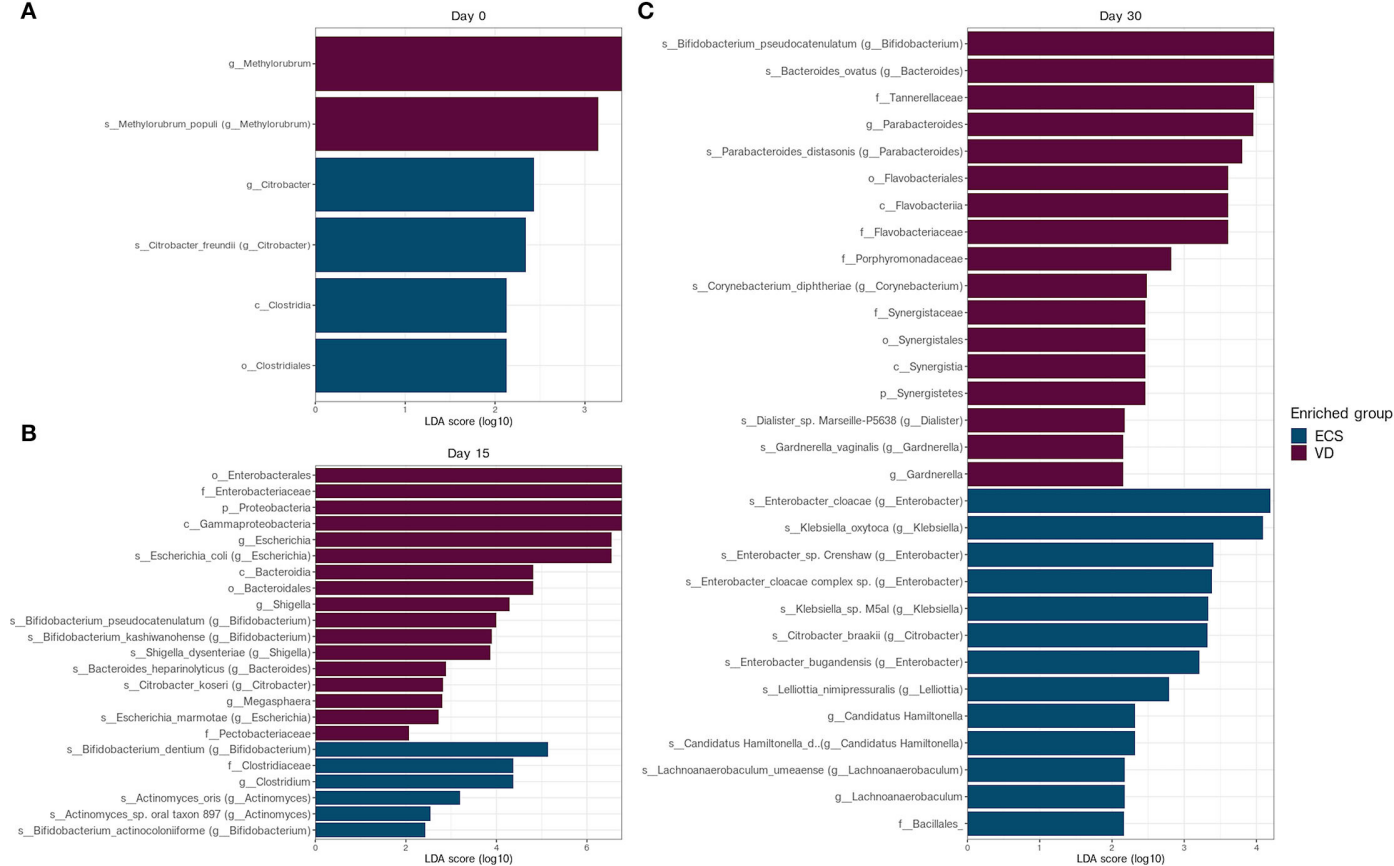
Differentially abundant taxa between ECS and VD newborns at each collection data. Linear discriminant analysis effect size (LEfSe) analysis of metagenomic sequences from ECS and VD newborn stool samples at Day 0 **(A)**, Day 15 **(B)**, and Day 30 **(C)**. The blue shaded bars indicate the taxa enriched in the microbiome of VD newborns. The purple shaded bars indicate the taxa enriched in the microbiome of ECS newborns. The prefixes “p,” “c,” “o,” “f,” “g,” “s,” and “s” indicate the annotation levels of phylum, class, order, family, genus, species, and strain, respectively.

Again, LEfSe analysis was used to detect overrepresented bacterial taxa between ECS and VD at each sampling time point. We found that few distinctive taxa signatures were detected in the neonatal microbiota at Day 0 (Figure 4A). While the Clostridiale order and the *Citrobacter* genus were overrepresented in ECS, the *Methylobacter* genus was detected in VD.

On Day 15, Proteobacteria, especially *Escherichia* and *Shigella*, were overrepresented in VD newborns compared with the ECS group (Figure 4B). The ECS group had a higher relative abundance of *Clostridium* and *Clostridiaceae*. In addition, the ECS group was enriched in species belonging to the *Bifidobacterium* genus, namely *B. dentium* and *Bifidobacterium actinocoloniform* (Figure 4B).

On Day 30, LEfSe analysis revealed an overrepresentation of species belonging to *Bacteroides, Parabacteroides*, and *Gardnerella* genera, respectively, represented by *Bacteroides ovatus, Parabacteroides distasonis*, and *Gardnerella vaginalis*, respectively, for the VD group (Figure 4C). The ECS samples showed an enrichment of *Klebsiella oxytoca* and various *Enterobacter* species (Figure 4C). Given the prevalence of these opportunistic pathogens in the gut of ECS neonates, we extended this analysis and compared the relative abundance of the ESKAPE group. We found that the ECS group had a significantly higher relative abundance at Day 15 and Day 30 (Supplementary Figure 4).

The above-mentioned results indicate that a shift occurs during the same timeframe in ECS and VD. However, ECS showed a disturbed establishment of the *Bacteroides* genus and an increased relative abundance of ESKAPE bacteria members from the second week.

## Discussion

This longitudinal study was designed to provide an unprecedented insight into the Tunisian newborn's gut microbiota using high-resolution shotgun sequencing. In our context of low biomass, this technique demonstrated its ability to better accurately assign reads to species level compared to 16s sequencing as stated by Peterson et al. (2021). It assessed the effect of DM, more precisely the ESC, on its composition during the first month of life. In fact, ECS represents a public health concern as this procedure is often over-practiced (Jenabi et al., 2020). Considering the establishment of newborn microbiota, we herein highlighted major differences between the day of birth and the other two time points in terms of alpha diversity. We observed the highest diversity at Day 0 compared with the other two time points. This trend is possibly due to the rapid influx of microbes composing the pioneering microbiome, originating from maternal and environmental origins, as previously suggested (Wampach et al., 2017; Ferretti et al., 2018). The subsequent decrease in diversity could be attributed to anterior loss or replacement of poorly adapted bacteria in the gastrointestinal tract (Ferretti et al., 2018). Similar to previous findings summarized by Milani et al. the Tunisian gut microbiota is initially colonized mainly by Proteobacteria, Actinobacteria, Firmicutes, and Bacteroidetes (Milani et al., 2017). At the genus level, this study indicates that the initial gut microbiota is dynamic and undergoes several changes in terms of composition starting from the second week onwards. This shift observed on Day 15 concerned particularly the genera *Klebsiella, Escherichia, Lactobacillus*, and *Staphylococcus*. These findings shade previous descriptions that suggested initial colonization by Firmicutes members such as *Streptococcus, Staphylococcus*, and *Enterobacteriaceae* (Rautava et al., 2012).

Early life microbiome colonization is influenced by numerous factors, and a particular emphasis has been placed on the impact of the mode of delivery. A considerable amount of literature has been devoted to this topic and has established that the gut microbiota of CS newborns is characterized by a reduced diversity and an altered composition (Dominguez-Bello et al., 2010; Shi et al., 2018; Stinson et al., 2018; Shao et al., 2019).

When considering the medical indication and the urgency of cesarean deliveries, previous analyses reported that infants born by ECS had the lowest richness and diversity compared with infants born by urgent cesarean delivery and those vaginally delivered (Azad et al., 2013). However, no significant difference was detected in terms of diversity between ECS and VD in the first days of life (Liu et al., 2015). Our results further support the latter observation, extending the observed trend to the first month of life. In addition, our results show a significant change in the composition of the microbiota during the first 2 weeks for the VD and ECS groups. These results are in agreement with those presented previously by Kim et al. The authors had also highlighted that this transition is progressive in VD, whereas it is abrupt for those delivered by CS (Kim et al., 2020).

Low species-specific diversity is commonly associated with a higher susceptibility to develop immune and metabolic diseases (Milani et al., 2017). Therefore, we sought to identify key taxa, highlighting this aspect. Interestingly, the *Bacteroides* genus was absent in ECS newborns, consistent with previous findings (Jakobsson et al., 2014; Shao et al., 2019). *Bacteroides* are acquired through exposure to maternal vaginal flora, thus explaining the low*-Bacteroides* profile for CS (Shao et al., 2019). Recently, Mitchell et al. challenged this dogma by demonstrating that *Bacteroides* were present in the first week and absent during the second week in both emergent and ECS newborns (Mitchell et al., 2020). The delayed establishment of *Bacteroides* in the gut microbiota of CS neonates was associated with health issues, notably allergic diseases, and milk oligosaccharides' breakdown (Marcobal et al., 2011; Jakobsson et al., 2014).

In the same context, previous studies considered the *Bifidobacterium* genus as the most abundant during the first days of life, with a lower prevalence in CS (Tanaka and Nakayama, 2017). In this study, we confirmed the high level of *Bifidobacterium* in VD compared with CS. In this frame, Yassour et al. suggested that the lack of *Bacteroides* is balanced by the abundance of *Bifidobacterium* (Yassour et al., 2016). Our differential analysis showed an enrichment of *B. dentium* and *Bifidobacterium actinocoloniiforme* in CS, while *B. pseudocatenulatum* and *Bifidobacterium kashiwanohense* are overrepresented in the VD group. Other studies described a delay in the transmission of maternal strains of *Bifidobacterium* (e.g., *Bifidobacterium longum, Bifidobacterium breve*, and *B. pseudocatenulatum*) in newborns delivered by cesarean (Shao et al., 2019). Moreover, Saturio et al. (2021) reported a higher abundance of *B. pseudocatenulatum* in the VD and *B. dentium* species in the CS.

Similar to *Bifidobacterium, Lactobacillus* is also considered a major beneficial bacterium for the health of the human host (Rastall, 2004). Its low prevalence was associated with increased susceptibility to allergic diseases (Wang et al., 2020). In fact, *Lactobacillus* is typically present in the maternal vaginal flora (Tannock et al., 1990; Chu et al., 2017) and is reported as enriched in VD newborns compared with CS newborns (Dominguez-Bello et al., 2010; Kim et al., 2020). However, it was reported that the presence of the *Lactobacillus* genus is not dependent on the urgency of CS (Liu et al., 2015). Our results showed that *Lactobacillus* is barely observable in VD, while undetectable in ECS.

Another striking result was the detection of an enrichment of opportunistic pathogens in ECS newborns. Among these pathogenic species, we particularly identified ESKAPE pathogen members, responsible for several nosocomial infections (Santajit and Indrawattana, 2016). Other highly opportunistic pathogens, namely *Klebsiella oxytoca, Enterobacter cloacae*, and *Clostridium perfringens*, were also retrieved as overrepresented in ECS (Shao et al., 2019). Using lower resolution methods, prior work showed a similar pathogen prevalence between ECS and VD, thus confirming our results (Liu et al., 2015; Stokholm et al., 2016). The prevalence of the latter pathogens in CS, and to a lesser extent in VD, was associated with *Bacteroides* alteration and non-breastfeeding practices (Shao et al., 2019). Thus, the observed low-*Bacteroides* profile could partially explain the dominance of ESKAPE bacteria in ECS at Day 15 and Day 30. Regarding breastfeeding practices, we could not assess its protective effect due to the small size of the groups.

In conclusion, this study provides the first overview of the ethnically homogenous and still unresolved microbiota of Tunisian newborns. Using a higher resolution Shotgun sequencing approach, we complemented previous findings regarding the impact of ECS on the initial colonization of the gut microbiota.

## Data Availability Statement

The datasets presented in this study can be found in SRA repository at: https://www.ncbi.nlm.nih.gov/bioproject/PRJNA762668.

## Ethics Statement

The studies involving human participants were reviewed and approved by Comité d'éthique bio-médicale de l'institut Pasteur de Tunis. Written informed consent to participate in this study was provided by the participants' legal guardian/next of kin.

## Author Contributions

OS and AB conceived this study and were in charge of overall direction and planning. MH, OM, HB, FZ, MBa, LG-T, FT, and OM assisted in the implementation of collection and DNA extraction protocols. MH, OS, LG-T, AB, MBe, and EH-S contributed to the interpretation of the results and provided critical feedback, and helped shape the research, analysis, and manuscript. MH, OS, AB, and LG-T analyzed the data and wrote the manuscript. All authors contributed with valuable discussions and editions and approving the final version of the manuscript.

## Funding

This project was partly funded by H3ABioNet, which was supported by the National Institutes of Health Common Fund (Grant No. U41HG006941). Financial support was also afforded by the foundation Mérieux. We would like to acknowledge the European project PHINDaccess: Strengthening Omics data analysis capacities in pathogen-host interaction (Grant Agreement ID: 811034).

## Conflict of Interest

The authors declare that the research was conducted in the absence of any commercial or financial relationships that could be construed as a potential conflict of interest.

## Publisher's Note

All claims expressed in this article are solely those of the authors and do not necessarily represent those of their affiliated organizations, or those of the publisher, the editors and the reviewers. Any product that may be evaluated in this article, or claim that may be made by its manufacturer, is not guaranteed or endorsed by the publisher.

## Supplementary Material

Supplementary Figure 1 Impact of feeding mode on newborn's gut microbiota. Alpha diversity was measured by estimating the Shannon index (panel top right), Simpson index (panel top left), observed OTU richness (panel bottom left), and Faith phylogenetic diversity (panel bottom right). Significant differences between groups were determined using the Kruskal–Wallis test, followed by the Dunn test with Bonferroni correction.

Supplementary Figure 2 Differentially abundant taxa between elective cesarean section (ECS) and vaginally delivered (VD) newborns detected by linear discriminant analysis effect size (LEfSe) analysis. The blue shaded bars indicate the taxa enriched in the microbiome of VD newborns. The purple shaded bars indicate the taxa enriched in the microbiome of ECS newborns. The prefixes “p,” “c,” “o,” “f,” “g,” “s,” and “s” indicate the annotation levels of phylum, class, order, family, genus, species, and strain, respectively.

Supplementary Figure 3 Bacterial diversity transition in ECS and VD newborns. Nonmetric Multidimensional Scaling (NMDS) ordination plot based on the relative abundance of taxa in VD **(A)** and ECS **(B)**.

Supplementary Figure 4 Relative abundance of ESKAPE members in ECS and VD newborns at each collection time point. Significance in the differences between groups was determined using the Mann–Whitney *U*-test.
