# Supplementary figures and images for: Longitudinal and Comparative Analysis of Gut Microbiota of Tunisian Newborns According to Delivery Mode
Source: Front Microbiol. 2022 Apr 25;13:780568. doi: 10.3389/fmicb.2022.780568 (PMC9083410; doi:10.3389/fmicb.2022.780568)

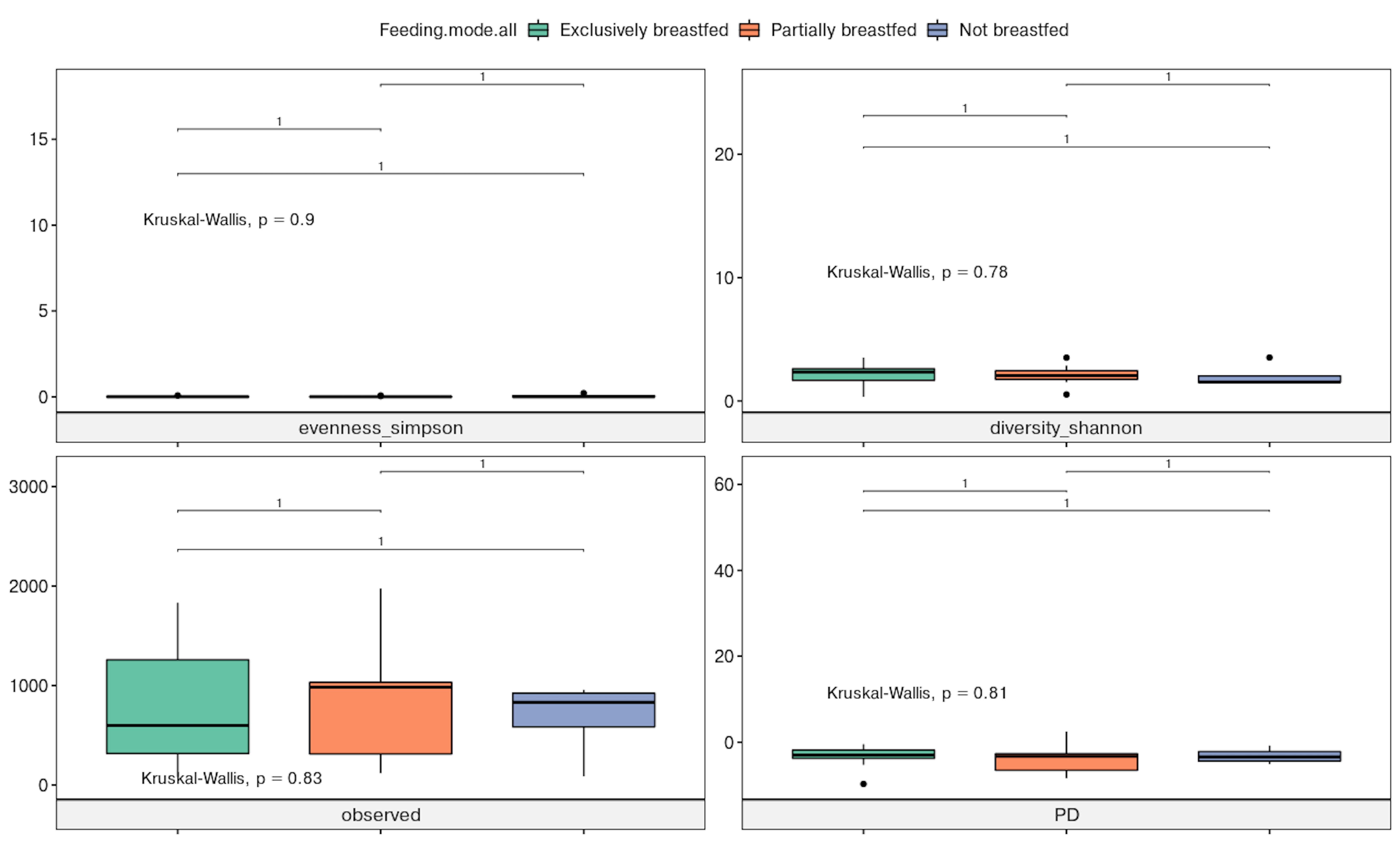

Supplement: Supplementary Figure 1 — Impact of feeding mode on newborn's gut microbiota. Alpha diversity was measured by estimating the Shannon index (panel top right), Simpson index (panel top left), observed OTU richness (panel bottom left), and Faith phylogenetic diversity (panel bottom right). Significant differences between groups were determined using the Kruskal–Wallis test, followed by the Dunn test with Bonferroni correction. [file Image_1.jpeg]

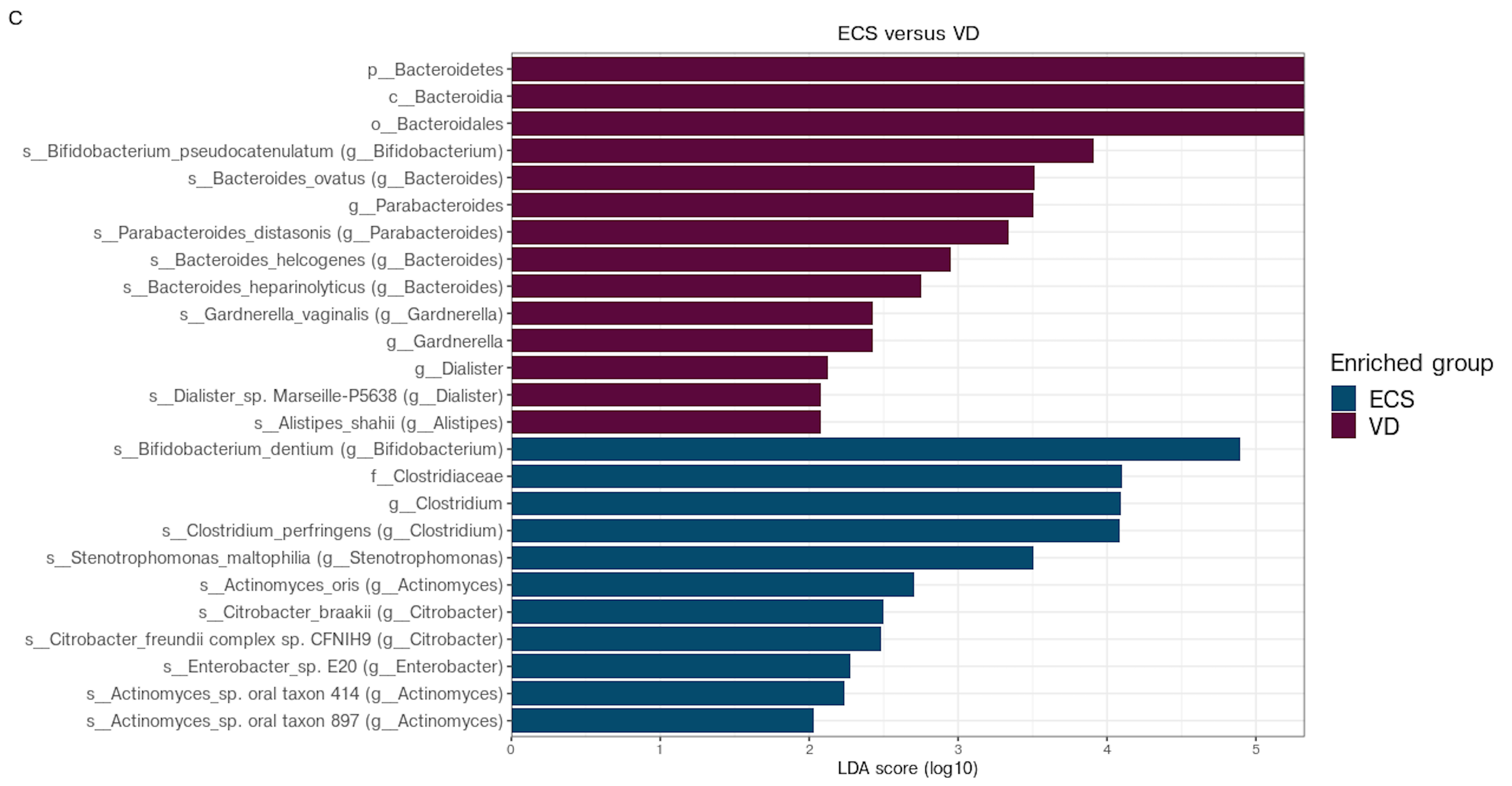

Supplement: Supplementary Figure 2 — Differentially abundant taxa between elective cesarean section (ECS) and vaginally delivered (VD) newborns detected by linear discriminant analysis effect size (LEfSe) analysis. The blue shaded bars indicate the taxa enriched in the microbiome of VD newborns. The purple shaded bars indicate the taxa enriched in the microbiome of ECS newborns. The prefixes “p,” “c,” “o,” “f,” “g,” “s,” and “s” indicate the annotation levels of phylum, class, order, family, genus, species, and strain, respectively. [file Image_2.jpeg]

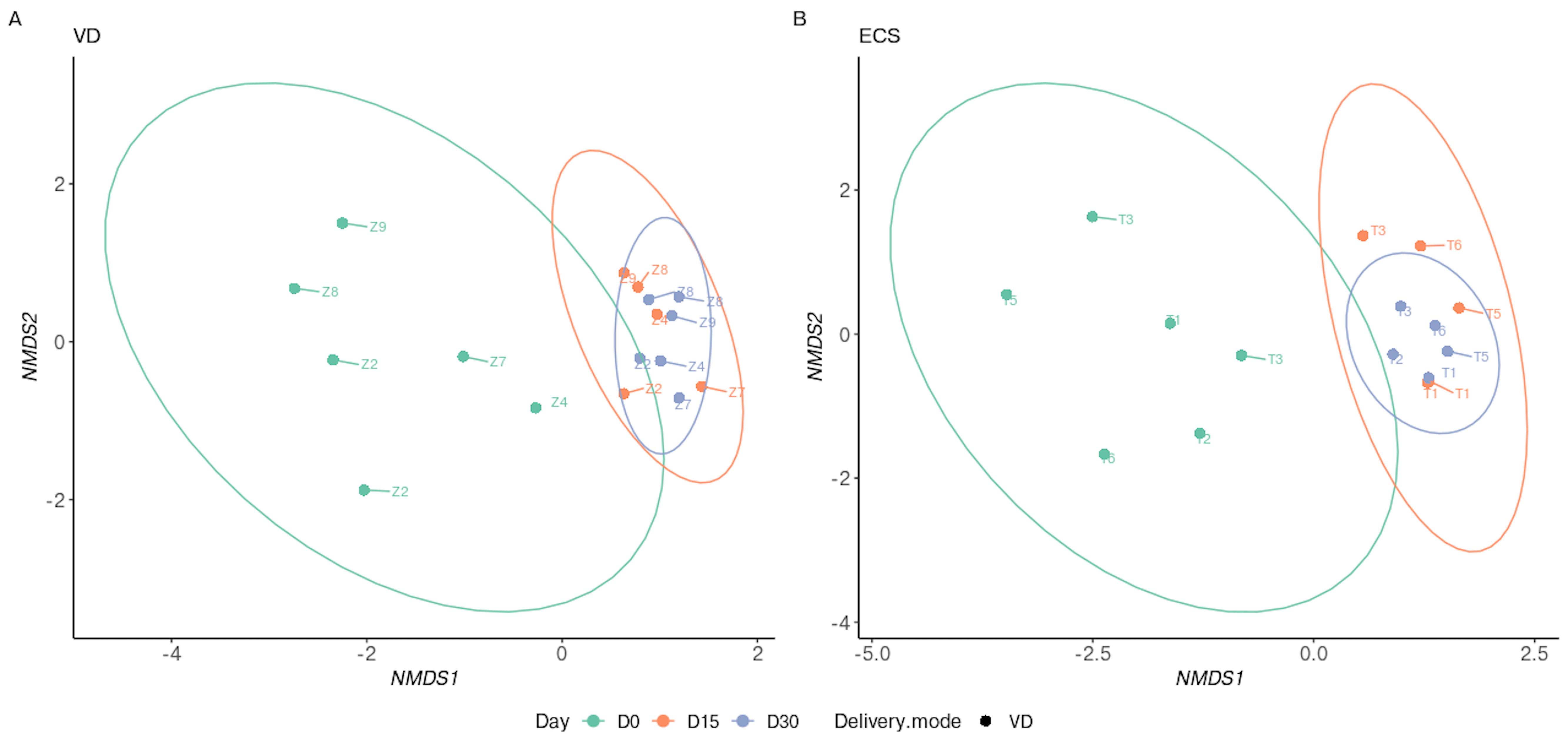

Supplement: Supplementary Figure 3 — Bacterial diversity transition in ECS and VD newborns. Nonmetric Multidimensional Scaling (NMDS) ordination plot based on the relative abundance of taxa in VD (A) and ECS (B). [file Image_3.jpeg]

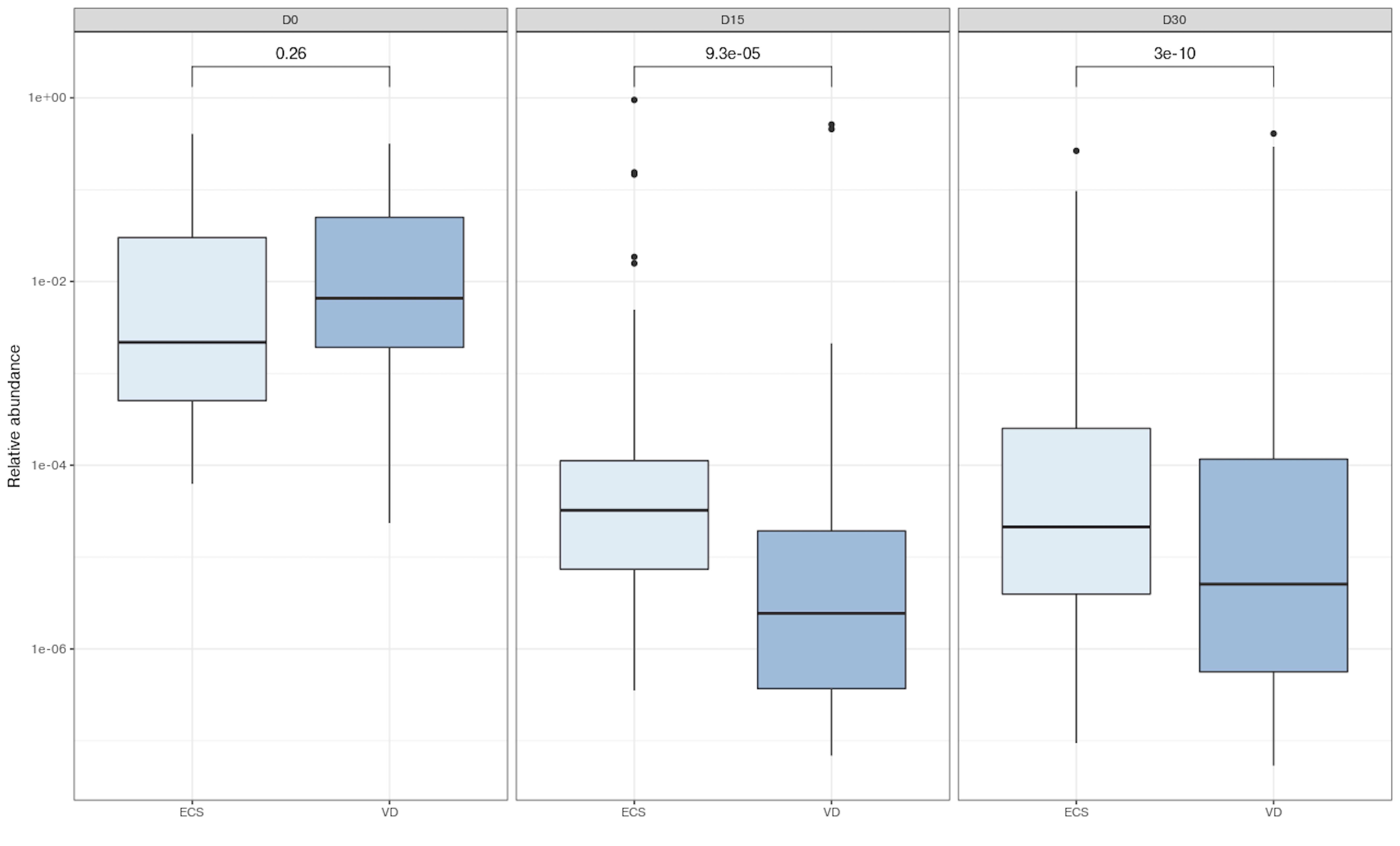

Supplement: Supplementary Figure 4 — Relative abundance of ESKAPE members in ECS and VD newborns at each collection time point. Significance in the differences between groups was determined using the Mann–Whitney U-test. [file Image_4.jpeg]
